# Quantum-resistance in blockchain networks

**DOI:** 10.1038/s41598-023-32701-6

**Published:** 2023-04-06

**Authors:** Marcos Allende, Diego López León, Sergio Cerón, Adrián Pareja, Erick Pacheco, Antonio Leal, Marcelo Da Silva, Alejandro Pardo, Duncan Jones, David J. Worrall, Ben Merriman, Jonathan Gilmore, Nick Kitchener, Salvador E. Venegas-Andraca

**Affiliations:** 1 IDB-Inter-American Development Bank, 1300 New York Ave, Washington, DC USA; 2 LACChain-Global Alliance for the Development of the Blockchain Ecosystem in LAC, Washington, DC USA; 3 Quantinuum, London, UK; 4 Tecnologico de Monterrey, Escuela de Ingenieria y Ciencias, Monterrey, NL Mexico

**Keywords:** Quantum information, Computer science, Information technology

## Abstract

The advent of quantum computing threatens blockchain protocols and networks because they utilize non-quantum resistant cryptographic algorithms. When quantum computers become robust enough to run Shor’s algorithm on a large scale, the most used asymmetric algorithms, utilized for digital signatures and message encryption, such as RSA, (EC)DSA, and (EC)DH, will be no longer secure. Quantum computers will be able to break them within a short period of time. Similarly, Grover’s algorithm concedes a quadratic advantage for mining blocks in certain consensus protocols such as proof of work. Today, there are hundreds of billions of dollars denominated in cryptocurrencies and other digital assets that rely on blockchain ledgers as well as thousands of blockchain-based applications storing value in blockchain networks. Cryptocurrencies and blockchain-based applications require solutions that guarantee quantum resistance in order to preserve the integrity of data and assets in these public and immutable ledgers. The quantum threat and some potential solutions are well understood and presented in the literature. However, most proposals are theoretical, require large QKD networks, or propose new quantum-resistant blockchain networks to be built from scratch. Our work, which is presented in this paper, is pioneer in proposing an end-to-end framework for post-quantum blockchain networks that can be applied to existing blockchain to achieve quantum-resistance. We have developed an open-source implementation in an Ethereum-based (i.e., EVM compatible) network that can be extended to other existing blockchains. For the implementation we have (i) used quantum entropy to generate post-quantum key pairs, (ii) established post-quantum TLS connections and X.509 certificates to secure the exchange of information between blockchain nodes over the internet without needing a large QKD network, (iii) introduced a post-quantum second signature in transactions using Falcon-512 post-quantum keys, and (iv) developed the first on-chain verification of post-quantum signatures using three different mechanisms that are compared and analyzed: Solidity smart-contracts run by the validators for each transaction, modified EVM Opcode, and precompiled smart contracts.

## Introduction

Quantum computing, one of the most recent cross-pollination efforts between physics and computer science, is a scientific and engineering field focused on developing information processing devices and algorithms based on quantum mechanics^1–7^. Quantum computing is now an established research field with solid theoretical and experimental results^8–14^. Furthermore, high-tech businesses across various sectors are increasingly experimenting with quantum computing technological solutions^15–18^.

Since the early days of quantum computing, the role of quantum algorithms and quantum protocols in information security has been a crucial issue. On the one hand, Shor’s algorithm^19^ could be used to break public-key cryptography protocols. On the other hand, Quantum Key Distribution schemes provide security levels to information transmission that are not based on mathematical conjectures but instead on the properties of quantum mechanics^20^. Quantum technology is expected to have a relevant role in current and future cybersecurity systems and, consequently, a significant impact on regional and global economic development^21^.

Quantum entropy provides perfect randomness and strong cryptographic keys based on quantum mechanics^22^. Post-Quantum Cryptography encompasses a new generation of algorithms for the creation of asymmetric keys that are thought to be resistant to attacks by quantum computers^23^.

Currently, blockchain^24^ is the most popular technology amongst emerging applications for decentralized data sharing and storage. The design and implementation of blockchain networks makes extensive use of cryptography protocols; thus, studying the potential uses of quantum computing and quantum information to both weaken and strengthen blockchain technologies is essential to ensuring its future reliability.

The rest of this paper is divided as follows. “Context” presents an introductory review of Quantum Computing, Quantum Key Distribution, Post-Quantum Cryptography, blockchain, and the LACChain Blockchain Network which was used for the implementation; “The vulnerabilities of blockchain technology with the advent of quantum computing” analyzes relevant vulnerabilities of blockchain within the context of quantum computing technologies; “Literature review” presents a detailed review of the state-of-the-art in the field; “Results I—our proposal for post-quantum blockchain networks” introduces our proposal for guaranteeing quantum-resistance in EVM compatible blockchain networks and describes the implementation carried out in the LACChain Blockchain Network; “Results II—our implementation in the EVM-compatible LACChain blockchain”, we present an implementation of our end-to-end quantum resistant blockchain network proposal based on the framework presented in “Results I—our proposal for post-quantum blockchain networks”; finally, on “Discussion” we present a discussion on the conclusions and future directions.

## Context

### Quantum computing as a threat to cryptography

Theoretical results, such as Shor’s algorithm^19^, and state-of-the-art quantum computing technology in conjunction with expected near-to-mid future scalability and robust developments, have attracted the attention of international standards agencies in cyber security and cryptography, including NIST^25^, NSA^26^, and ETSI^27^. These organizations have made critical warnings that running some quantum algorithms on full-scale quantum computers will necessitate the protection of internet and telecommunication information exchanges for widely used cryptography protocols. Most notably, NIST has been running a post-quantum cryptography competition for standardization to replace existing cryptographic algorithms that are susceptible to breakage using quantum computers^28^. On July 5th 2022, the first round of candidates to be standardized was published^29^.

Quantum computers use quantum bits (qubits) as fundamental units of information. Individual qubits can be in binary zero and one states (classical bits), but they can also be in any state between zero and one, which is defined by the superposition $\alpha |0\rangle + \beta |1\rangle$ where $\alpha , \beta \in {\mathbb {C}}$ subject to $|\alpha |^2 + |\beta |^2 =1$. Qubits leverage quantum effects that do not appear in classical computing, such as quantum superposition, quantum entanglement, and quantum tunneling. These effects are fundamental for the development of quantum algorithms, which have proven to be very useful in solving certain problems much more efficiently than the best-known classical algorithms, such as optimization or prime factorization of integer numbers.

In general, physical channels currently used to transmit digital information are unprotected (e.g., optical fibers or wireless transmissions) and the security of data exchanges within these channels relies on cryptographic protocols. It is only a matter of time before large and robust quantum computers capable of breaking current cryptographic protocols are built. It is crucial that we be prepared for these future technologies, especially in order to investigate the transition to quantum-safe cryptography for blockchain technologies.

### Current approaches for quantum-safe cryptography

Discussions on quantum computers and cryptography usually surround two main areas of cryptography that are thought to resist attacks by large and robust quantum computers: quantum key distribution and post-quantum cryptography.

#### Quantum key distribution

Quantum Key Distribution (QKD) refers to quantum protocols for the co-creation of private symmetric keys between two parties using quantum and classical channels (e.g., optical fibers and wireless channels) for codifying private key bits into quantum states. If these quantum states are intercepted and observed by any eavesdropper, the information they contain (i.e., the bits of the key) is modified, and therefore the key is corrupted and the eavesdropper is detected. Best known QKD protocols are BB84^30,31^ and E91^32^.

An illustrative example of a QKD implementation is the BB84 protocol using polarized photons. In this protocol, we have a sender (Alice), a recipient (Bob), and an eavesdropper (Eve). Alice codes the bits of a private key to share with Bob using non-orthogonal quantum states, such as bit value 0 using either $|0 \rangle$ or $|+ \rangle$ and bit value 1 using $|1 \rangle$ or $|- \rangle$. Then, photons are sent by Alice to Bob. Due to the properties of measurement in quantum mechanics, Eve’s eavesdropping activities will eventually be detected (that is, Eve’s activities will leave a trace that will eventually be detected by Alice and Bob) and, consequently, the protocol will stop and start over at a later stage^33,34^.

QKD protocols such as BB84 and E91 have been successfully implemented since 2003. However, QKD is not fully scalable today because ground-based key exchanges using optical fibers are limited to a few hundreds kilometers due to the degradation of the quantum states containing the keys^35^. Additionally, ground-to-satellite key exchanges require sophisticated infrastructure for generation, transmission, and reception of quantum keys^36,37^. The scalability of these networks depends on the development of quantum repeaters, which require very sophisticated quantum memories. This is still an area under development^38,39^. For these reasons, QKD has been discarded as a feasible solution to provide quantum safeness to blockchain networks today. However, this may change in the future as NSA, NIST, and ETSI, among others, have declared that quantum cryptography (such as QKD) would be the only alternative for long term secure encryption^25–27^.

#### Post-quantum cryptography

The most popular asymmetric cryptography schemes used today are believed to be vulnerable against quantum adversaries. These include RSA^40,41^, (Elliptic Curve) Digital Signature Algorithm^42^, and (Elliptic Curve) Diffie-Hellman^43,44^.

Post-quantum cryptography (PQC) refers to a new generation of asymmetric algorithms that cannot be broken by Shor’s algorithm and therefore are considered quantum-resistant. Unlike QKD, PQC does not rely on any underlying quantum processes for the exchange of symmetric key pairs but rather on leveraging mathematical problems more complex than the existing ones for the generation of asymmetric keys. The main focus areas for post-quantum algorithms to generate quantum-safe asymmetric key pairs are:

- Hash-based cryptography, based on the security of hash functions.
- Code-based cryptography, based on the difficulty of decoding generic linear code.
- Lattice-based cryptography, based on the difficulty of well-studied lattice problems (e.g., shortest vector problem).
- Multivariate cryptography, based on multivariate polynomials over a finite field.

As mentioned above, there is a standardization process being conducted by NIST which started in August 2016 with a request for candidates for post-quantum cryptographic algorithms^28^. This process, which called for submissions in the areas of “Public-key Encryption and Key Establishment Mechanisms (KEM)” and “Digital Signature Algorithms” announced the final and alternate rounds of in July 2020^45^.

The Candidates to be Standardized and Round 4 Submissions were announced on July 2022^29^. The results, detailed in NISTIR 8413 Status Report^46^, can be summarized as follows:

NIST’s selected algorithm in the KEM category is:

- Crystals-Kyber, a suite of algebraic lattices utilizing a Kyber primitive for KEM^47^.

NIST’s selected algorithms in the Digital Signature category are:

- Crystals-Dilithium, a suite of Algebraic lattices using a Dilithium primitive for signature^48^.
- Falcon, lattice-based algorithm with shake256 hashing^49^.
- SPHINCS+, stateless hash-based signature scheme^50^ .

Additionally, NIST also announced four candidates for PQC Standardization Round 4, all of them in the KEM category: Classic McEliece^51^, BIKE^52^, HQC^53^, and SIKE which was defeated later in August using a classical computer and removed from the list^54^.

### Blockchain, ethereum, and the LACChain blockchain network

Blockchain is a technology that allows one to build decentralized ledgers in which different entities can register transactions that are grouped into blocks that are linked using hashes^24^. The immutability of the transactions stored in blockchain networks is guaranteed because it is impossible to tamper with the ledger without being detected. As any entity can, in principle, have a synchronized copy of the ledger and transactions are validated according to predefined rules, the history cannot be rewritten. The integrity of the transactions is guaranteed by digital signatures because every transaction is signed by the sender, and the immutability of the chain is guaranteed by hash functions^24^.

Blockchain can be thought of as a computational system with a distributed state shared among a network of nodes, of which consistency can be verified by any participant. The state is dynamically updated through transactions that are broadcasted by the nodes, and each participant can have a verified and verifiable copy of the state and the transaction history. These transactions allow users to deploy executable code to the network, a.k.a. smart contracts, and interact with them.

In order for a new state to be agreed upon by the network, a subset of nodes, called validator or producer nodes, apply a consensus protocol. There are different types of consensus protocols and each network decides which type of consensus protocol they implement. Essentially, every consensus protocol consists of a set of rules that establish how these nodes will accomplish a computational validation of the latest transactions replicated across the network. The validator or producer node proposes a package, called a block, which contains the transaction, block number, nonce, block hash, previous block hash, and signatures of the block validators or producers that have validated the block. With this, a new block is cryptographically sealed and, once appended to the blockchain, it cannot be undone or tampered with.

In Ethereum Networks, the code deployed in the network is a stream of bytes representing operation codes from the Ethereum Virtual Machine (EVM). This set of operations can be considered Turing complete and are executed as a stack machine with a depth of 1024 items. The EVM is then the runtime environment where any state transformation takes place^55^. Every smart contract has its own memory space and can be changed or updated by a transaction, which is recorded in the transaction history and implies a modification of the current distributed state. Additionally, each operation has an associated cost, which is an abstraction of the computational power required to perform the requested action by an ideal computer. The cost is called gas and serves as a metric for the amount of computation required to process each block.

There are hundreds of EVM compatible blockchain networks. A non-exhaustive list of the most prominent permissionless ones is provided by Chainlink^56^. The Ethereum community is known to be the largest blockchain community in terms of both developers and users. There are hundreds of billions of dollars in assets relying in these networks in the form of cryptocurrencies, NFTs, and applications on top, among others. Ethereum Mainnet, the first EVM compatible Mainnet which was launched back in 2015, reached a historical maximum of 569 billion dollars market cap for its native cryptocurrency Ether in November 2021^57^.

If we add to that the value of every other asset and application running on top of the network, it is straightforward to foresee that not protecting these networks against quantum adversaries could lead to a very critical global financial crisis.

The solution we have developed for EVM compatible blockchain networks, which is described in “Results I—our proposal for post-quantum blockchain networks”, has been implemented and tested in the LACChain blockchain network. LACChain is a blockchain infrastructure led by the Innovation Lab of the Inter-American Development Bank (IDB Lab) in Global Alliance with some of the entities leading the development of blockchain technology in the world^58^. By the end of 2022, LACChain has become the largest permissioned public blockchain infrastructure in the world with 80+ projects and 200+ entities running nodes^58^. LACChain was built using Hyperledger Besu which is an Ethereum client originally developed by Consensys and now maintained by the Hyperledger and Ethereum communities, including Consensys^59^. LACChain was chosen for the implementation and evaluation of the solution for several reasons, among them:

- One of the teams involved in this project was the architecture team of LACChain, which encompasses experts in blockchain and quantum technologies.
- By having the LACChain team involved, we optimized deployment scripts and tools to run networks and nodes and monitor their activity in real time. This facilitated implementing the new protocols for communicating nodes and verifying post-quantum signatures, while monitoring results in real time.
- The fact that the LACChain blockchain infrastructure is used by several governments, banks, multilaterals, universities, and private sector companies for a large number of projects makes its capacity to resist attacks by quantum computers of high importance.
- The solution is compatible with other EVM blockchain networks, including Ethereum Mainnet. Therefore, using this network from the long list of EVM compatible networks was a very convenient decision based on the reasons detailed above and did not limit the scope of proposing an EVM compatible quantum-resistant solution, agnostic to the specific Ethereum-based blockchain protocol or network used for the implementation.

LACChain is one of the largest blockchain networks in the world in terms of identified institutions and projects using it. The list of entities includes the World Bank, Citi Bank, Banco Davivienda, Central Bank of Colombia, Brazilian Development Bank, Inter-American Development Bank, custom administrations of 8 LAC countries, the Chamber of Commerce of Lima, World Data, NTT Data, Tata Consulting Group, Izertis, Extrimian, and many others^60–66^. More than 80+ enterprise projects are taking place in the LACChain Network including large projects in the areas of health certificates, diplomas, bonds, procurement, digital identity, and traceability of supply chains. Securing assets and projects happening in this network is of vital importance.

## The vulnerabilities of blockchain technology with the advent of quantum computing

The advent of quantum computing constitutes a new paradigm in which digital technologies will endure both challenges and opportunities. Threats will come up in a variety of forms, especially when robust quantum computers will be able to break several important cryptographic algorithms currently used. Blockchain, as a technology that strongly relies on cryptography, is not safe from these threats. As stated in the literature^67–69^, it is worth exploring the conjunction of blockchain technology and quantum computing in the following five areas:

- Digital signatures are one of the most essential components of blockchain technology. Bitcoin and Ethereum use elliptic curve cryptography (ECC), particularly the ECDSA signature schemes on curve secp256k1. Others, such as EOSIO, use the NIST standard secp256r1 curve. NIST recommends that ECDSA and RSA signature schemes be replaced due to the impact of Shor’s algorithm on these schemes^70^.
- Communication over the Internet relies on protocols such as HTTP. The security of the communication happens in HTTPS within the SSL/TLS protocol stack. TLS supports one-time key generation with AES for symmetric encryption and several non-quantum-safe algorithms for exchange and authentication, such as RSA, DH, ECDH, ECDSA, and DSA. This means that all internet communications, including transactions and messages sent between applications and nodes in a blockchain, will not be quantum safe when robust quantum computers become fully operational.
- Block mining. Blockchain networks that use proof-of-work as the consensus mechanism rely on finding nonces. Quantum computers will be able to find these nonces quadratically faster using Grover’s algorithm^71^. However, this does not pose a major threat to the security of blockchain networks because the solution will be as easy as quadratically increasing the difficulty to compensate for the quantum advantage. In networks with consensus protocols that do not promote competition between nodes, such as the proof-of-authority used in the LACChain Blockchain, this threat will not exist.
- Reverting hashed data. Hash functions take an element from a set of infinitely many elements and gives an output from a finite set of $2^{256}$ elements in the case of the SHA-256 function that is used by most of the blockchain networks today. Thus, from a hash value stored in the blockchain, it is statistically impossible to obtain the element that resulted in that value. This property, known as pre-image resistance, guarantees that data stored in the blockchain in the form of hashes will remain undecipherable even in the presence of quantum computers which is essential for applications such as notarization.
- Rewriting history. Grover’s algorithm^71^ quantum advantage for nonce finding could provide a quadratic advantage to rewrite blocks changing the data and maintain the hashes, and therefore remaining undetected. It is yet unclear if this advantage could be sufficient to pose a threat for several reasons. Firstly, in order for a quantum adversary to rewrite past blocks data and generate the same block hashes in a valid way, they would need to have also discovered the private keys of all the accounts they want to hack when rewriting transactions. Therefore, if we solve the problem of hacking accounts and assets, this is prevented. Secondly, blockchain networks have a certain finality; when rewriting past blocks and proposing a new version of the chain to the other nodes, even if the hashes of all modified past blocks match the original hashes, the new current state needs to match also the previous current state. Different blockchain networks might allow minor discrepancies depending on the finality of the network but in general it would lead to nodes refusing the rewritten history version of the chain. Thirdly, it is unclear how fast a quantum computer could be rewriting histories consider the difficulty of the problem and the fact that Grover’s algorithm only provide quadratic advantage. Additionally, hash functions are continually evolving for increased security. For example, if quantum computers evolve to the point of posing a threat to SHA-2, then SHA-3 is already standardized as an alternative that offers a higher level of security in NIST standard FIPS202^72^.

## Literature review

The quantum threat to current cryptography has been widely acknowledged since NIST^25^ and NSA^26^ 2016 reports. The blockchain ecosystem is aware of this threat, and leaders such as Vitalik Buterin, one of the founders of the Ethereum blockchain technology, stated back in 2013 when addressing an audience that “if you have bitcoins in an address you never use they are safe. Otherwise, anyone can steal them”^73^ and suggested in 2015 moving towards Lamport signatures eventually^74^. However, in July 2022, Vitalik shared in the Eth 2.0 conference that there is not yet a plan or roadmap for Ethereum to become quantum-resistant because the problem is being postponed to solve more urgent matters such as scalability, interoperability, or costs until quantum computers are ready^75^.

This is the most common standpoint across the blockchain community. Despite the awareness of the advent of quantum computers, there is not a feeling of urgency because there are more urgent challenges to be addressed. Neither there is, in general, a full understanding of the implications that the hacking capacities of quantum computers will have in blockchain networks. The topic is not even addressed in most of the most important blockchain conferences worldwide. However, more in the theoretical than in the experimental arena, there has been some interesting work that is worth reviewing.

The overview of the challenge that quantum computers represent for blockchain technology has been accurately covered in the literature^68,69,76^, aligned with the discussion that we presented in “The vulnerabilities of blockchain technology with the advent of quantum computing”. Some scientists have been developing models to predict the number of qubits necessary to break the cryptography of blockchain networks. Pioneer work by the University of Waterloo and Microsoft Research estimated that the number of logical qubits necessary to implement quantum algorithms that can break 256 bit-long digital signatures generated with (EC)DSA, typically used in current blockchain networks, are 1500^77^ and 2330^78^, respectively. It is still unclear how many physical qubits would be needed for that purpose. Another study by researchers in Singapore, Australia, and France claimed in 2017 that quantum computers would be large and robust enough to break Bitcoin keys in 10 minutes by 2027^79^. More recent work published in 2022 by M. Webber et al. claims that we would need $1.9 \times 10^9$ physical qubits to break the Elliptic Curve encryption of Bitcoin within 10 minutes, $3.17 \times 10^8$ physical qubits to break it within one hour, and $1.3 \times 10^7$ to break it within one day^80^.

In December 2022, a research group claimed to have optimized the factorization of prime numbers using quantum computers in a way that it would be possible to break RSA-2048 keys with a quantum circuit of 372 physical qubits and a depth of thousands and presented to have fully factorized the integer 261980999226229 (48-bit) using it, becoming the largest prime number to by factorized by a quantum computer to date^81^.

Some work of reference has been done in proposing solutions for blockchain networks and protocols to resist attacks by quantum computers. The proposals developed to date can be classified into two broad groups: quantum blockchain networks and post-quantum blockchain networks.

Quantum blockchain networks are those that leverage quantum phenomena to make blockchain networks quantum resistant, including QKD to protect the communication between nodes and entanglement in time to achieve no-cloning of transactions and therefore prevent double spent^82–86^. There are also research efforts that include the use of quantum circuits for decentralized asset exchanges^87^ and frameworks for quantum identity authentication^88,89^. The problem with these approaches is that they assume QKD channels between nodes are available. However, as discussed in “Quantum key distribution”, there is still a lot of challenges being addressed internationally to build large, robust, and scalable QKD networks. Therefore, quantum blockchain networks leveraging quantum communication protocols will have to wait for a global QKD-based Internet which still is a bit far away and cannot be counted on for short-term quantum-resistance.

Post-quantum blockchain networks can be defined as those leveraging post-quantum cryptography to ensure quantum-resistance. There is literature of reference for each of the four post-quantum families of algorithms presented in “Post-quantum cryptography”. For instance, QS-RP, a blockchain-based quantum-secure reporting protocol using the multivariate public-key cryptography is presented in^90^. Furthermore^91^, proposes an e-voting protocol based on blockchain that uses code-based cryptography to ensure quantum resistance. However, most of the work is focused on hash-based and lattice-based cryptography. A group of scientists developed the MatRiCT lattice-based quantum resistant protocol built on ring confidential transactions (RingCT) which is the protocol used by Monero cryptocurrency to hide transaction amounts^92^.

Li et al. implemented a lattice-based solution where public and private keys are generated with Bonsai Trees technology, and used algorithms that ensure randomness and construct lightweight nondeterministic wallets^93^. Regarding hash-based cryptography proposals, Suhail et al. present a very complete analysis of the state of the art with a focus on applications for IoT^94^. Another work carried out by R3, the company behind the permissioned decentralized ledgers Corda, proposes the BPQS scheme, which is claimed to outperform existing hash-based algorithms when a key is reused for reasonable numbers of signatures, while supporting a fallback mechanism to allow for a practically unlimited number of signatures if required^95^.

One more group presents an interesting approach for digital signature based on hash chains^96^. While these works on post-quantum blockchain are very promising, they are not providing end-to-end solutions for quantum-resistant blockchain networks, as these schemes are only for protecting digital signatures and assets. More importantly, with the exception of the MatRiCT protocol applicable to the Monero cryptocurrency, none of the other proposals are targeting specific existing blockchain networks. Therefore, there is not a direct takeaway for securing the current hundreds of billions of dollars in current assets stored in existing blockchain networks.

It is also worth discussing the case of IOTA, a decentralized ledger intended for the Internet of Things. IOTA is popular for implementing hash-based signatures, specifically the Winternitz one-time signature scheme^97^, and therefore be quantum resistant. However, IOTA is a direct acyclic graph (ACG) not a blockchain, as it is claimed in its own documentation^98^.

On a parallel note, because our proposal is based on a hybrid cryptosytem than combines classical and quantum cryptography, an analysis of prior work on this incipient area is very relevant. Transitioning from classical cryptography primitives to post-quantum ones is one of the biggest challenges that cryptography community faces today. Most of the post-quantum algorithms participating in NIST’s standardization project^45^ are relatively new and their adoption is still in their early days. Therefore, in order to achieve a swift transition and maintain strong security at the same time, a hybrid approach of combining classical and post-quantum algorithms has been proposed to several cryptographic applications.

In 2016, Google performed an experiment named CECPQ1^99^, to integrate post-quantum key exchange in TLS 1.2 handshake. CECPQ1 used a hybrid key exchange algorithm by combining X25519 ECDH with NewHope lattice-based key exchange^100^. This was later improved in the follow-up project CECPQ2^101,102^ in 2019 in collaboration with Cloudflare. This has led several other industry players to further develop hybrid key exchange protocols, such as Amazon^103^ and Mozilla^104^. In^105^, a group of researchers introduced hybrid post-quantum certificates by combining the classical ECDSA scheme with post-quantum signature schemes. Another group in^106^ investigated hybrid signature schemes focusing on fast signing speed. Recently, Crockett et al.^107^ published a survey on several case studies for post-quantum and hybrid schemes integration in TLS and SSH.

## Results I—our proposal for post-quantum blockchain networks

As a result of the discussion presented over the previous sections of this paper, it becomes clear that the threat blockchain networks face with respect to quantum computers is primarily related to vulnerable digital signatures of blockchain transactions and vulnerable key-exchange mechanisms used for the peer-to-peer communication over the network. Our proposal consists in a 5-step end-to-end framework applicable to most blockchain networks that allow to achieve quantum-resistance to communication, signatures, and assets. Our approach is post-quantum and therefore relies on quantum-resistant public key algorithms. It can be described as follows:

1. Generation and distribution of quantum entropy: Provide every node with a source of quantum entropy so post-quantum keys can be generated based on quantum pure randomness. If nodes cannot have their own, establish a quantum-resistant connection for quantum entropy to be provided from a central source.
2. Generation of post-quantum certificates: Have a Certificate Authority generating post-quantum X.509 certificates for the node owners using the post-quantum public keys generated using the local source of quantum entropy. These post-quantum X.509 certificates use the v3 extension specifications for X.509 certificates that allow to add custom cryptographic algorithms.
3. Encapsulation of the communication between nodes using quantum-safe cryptography: Create post-quantum TLS tunnels between nodes using the post-quantum X.509 certificates so all the communication between nodes (i.e., transactions to be broadcast or replicated and new blocks proposed by validator nodes) is quantum-resistant.
4. Signature of transactions using post-quantum keys: Adding a post-quantum signature to every transaction leveraging a new post-quantum algorithm agreed upon by the entire network. Every transaction without a post-quantum signature is to be ignored by every node. Post-quantum signatures prevent impersonations and asset hacking with quantum computers.
5. On-chain verification of post-quantum signatures: Efficient and scalable mechanisms to verify the post-quantum signatures on-chain.

Unlike other solutions discussed in “Literature review”, our framework is algorithm-agnostic. Our post-quantum approach is pioneer in using quantum entropy for the key generation and achieves quantum-resistance in the communication between nodes at a large scale without needing QKD networks which, as discussed in “Quantum key distribution” and “Literature review”, will not be ready for short- and mid- term global blockchain networks. Additionally, our proposal to adding a post-quantum signature allows to secure the billions of dollars in assets stored in existing blockchain networks without having to replace the underlying cryptographic algorithms, which is unfeasible for most existing blockchain networks. For the verification of the post-quantum signatures, we have been pioneer in developing three open-sourced mechanisms for EVM compatible (i.e., Ethereum-based) networks to make on-chain verifications. Our implementation and results are presented and discussed in “Results II—our implementation in the EVM-compatible LACChain blockchain”.

## Results II—our implementation in the EVM-compatible LACChain blockchain

In this section we present our development of an end-to-end quantum resistant blockchain network following our framework presented in “Results I—our proposal for post-quantum blockchain networks”. It is organized in five subsections that map the five steps of our framework.

In “Quantum origin platform”, we describe the use of Quantum Origin as a centralized entropy source. We also detail how the entropy is provided to every node using quantum-safe connections based on McEliece KEM keypair exchanges. Our use of quantum entropy is pioneer in the literature.

In “Generation of post-quantum certificates”, we describe how every node uses quantum entropy to generate Falcon keys and post-quantum X.509 certificates. To that purpose, every node uses a modified version of libSSL, and generates and sends a CSR to the Certificate Authority (CA). The CA verifies the node’s identity, issues the post-quantum X.509 certificate to them, and registers their identifier in the blockchain.

In “Encapsulation of the communication between nodes using quantum-safe cryptography”, we explain how nodes leverage their post-quantum X.509 certificates with Falcon-512 public keys to establish quantum-resistant TLS tunnels.

In “Signature of transactions using post-quantum keys”, we describe how nodes use their post-quantum Falcon-512 keys to sign every transaction they broadcast to the network, complementing the ECDSA native signature required by the blockchain protocol.

In “On-chain verification of post-quantum signatures”, we describe, compare, and analyze our pioneer implementation of three different on-chain verification mechanisms of Falcon-512 post-quantum signatures in EVM-compatible networks: Solidity smart-contracts run by the validators for each transaction, modified EVM Opcode, and precompiled smart contracts.

It is worth pointing out that we performed our implementation between NIST’s round 3 and round 4 submissions, when both McEliece and Falcon were considered as finalists. Later in June 2022, Falcon was finally selected and McEliece has remained under review as candidate. We follow NIST’s standardization process closely to utilize certified post-quantum algorithms according to the latest releases.

Our specific implementation has been deployed an tested in the LACChain Blockchain Network introduced in “Blockchain, ethereum, and the LACChain blockchain network” and can be replicated in other EVM compatible ledgers. For non-EVM compatible ledgers it would be necessary to develop a different mechanism to introduce the post-quantum signature and its verification.

### Quantum origin platform

Randomness is the cornerstone upon which cryptographic standards are built. It is used to generate the keys and seeds used in cryptographic schemes. The challenge related to the generation of randomness is the generation of truly random data. Current techniques rely on deterministic approaches—hardware utilizing classical physics, and any available inputs that might add some level of unpredictability—which leads to the generation of pseudo-random data in the vast majority of the cases. Failure to ensure sufficient randomness in cryptographic processes can lead to real-world attacks on otherwise secure systems. This even extends to quantum random number generators which is why there is a need to develop schemes for true randomness^108^.

Conversely, quantum generation of randomness harnesses the power of the non-deterministic nature of quantum mechanics. Generating quantum random numbers^109^ can be built in many ways, as has been illustrated by the various approaches used to date, including beam splitters with detectors, vacuum fluctuations in coherent light, and squeezed coherent light mechanisms, among others^110,111^. Despite the fact that these methods are non-deterministic, they lack the ability for an end user to guarantee that the device is working correctly. This ability in a device (sometimes known as device independence or more commonly, as certifiably quantum generation) is at the heart of the qRNG, Quantum Origin, used in our solution presented in this paper.

Quantum origin generates randomness through a quantum process evaluated as quantum verifiable which utilizes a test for the violation of a Bell Inequality^112,113^ or a higher order test of a Mermin Inequality on a NISQ machine^114^. Such a violation, along with various other security tests, are taken as mathematical proof that the output could have only come from a quantum source and is non-deterministic and thus maximally random for a physical system. For the experiments in this paper, a quantum computer was used to generate the entropy.

Given the distributed nature of a blockchain, ideally each entity running a node should have its own local source of quantum entropy: a qRNG device. However, it was not feasible to provide each node with its own qRNG for our pilot, so we used a central source of quantum entropy. As discussed throughout this paper, current cryptographic schemes used in SSL/TLS are not quantum-safe, so using them to distribute the entropy would have broken the quantum-safeness at the start.

We decided instead to design a protocol that allowed nodes to create a quantum safe tunnel between themselves and the entropy distribution point to ensure that this communication could be considered quantum safe. In order to do this, the entropy source creates a first key, splits it into several parts, and delivers it to the node through various TLS channels. Nodes have a time out to receive the key, recompose it, and use it to authenticate against the entropy source. This is covered in more detail in “Entropy source setup”.

#### OpenSSL framework

Over the last 20 years, the OpenSSL API has become the de-facto cryptographic framework for applications that use TLS/SSL, providing capabilities such as:

- Generation of pseudo-random numbers.
- Classical cryptographic support using algorithms such as Diffie–Hellman (DH) and elliptic curve Diffie–Hellman (ECDH).

The OpenSSL applications and libraries also provide the following functions:

- Generation of private and public key pairs.
- Certificate authority management.
- Certificate validation.
- Management of crypto libraries and engine plugins to support new algorithms.
- SSL/TLS client and server implementations.

Because quantum computing will impact the security of asymmetric cryptographic algorithms such as RSA and ECDSA, the following changes within OpenSSL are required:

- Support for certified quantum entropy to replace the existing pseudo-random number generator used to seed keys and random values used for nonce parameters.
- Support for post-quantum algorithms to provide both key encapsulation and digital signatures.

Quantum origin platform facilitates the move to OpenSSL with entropy provided for:

- Quantum key encapsulation protecting existing PKI infrastructure by wrapping non-post quantum resistant keys in a post quantum wrapper.
- Quantum generated random numbers for pure quantum generated keys for signature digest algorithms.

This approach facilitates easy integration into computer security layers within the operating system while still being compatible with most of the existing infrastructure. The Quantum Origin) Service Agent provides post quantum encapsulated key management for the secure entropy tunnel back to the Quantum Origin platform. The component provides users with the ability to enforce customer security policies with regard to maximum key lifetimes by automatically providing configurable key cycling capability.

#### Entropy source setup

As mentioned before, every blockchain node should ideally have its own source of quantum entropy. For our pilot, LACChain nodes did not have a local source of quantum entropy so it was necessary to establish a quantum-safe connection between the external source (the Quantum Origin Platform) and each of the nodes. As the quantum entropy is necessary to generate the post-quantum keys that allow establishment of a quantum-safe connection, we could not use post-quantum cryptography to protect this first channel.

Therefore, we designed a protocol that begins with the distribution of a post-quantum key from the Quantum Origin Platform to the LACChain nodes. This key is split into *N* parts and delivered through different TLS channels. Once the LACChain node is in possession of all *N* parts, it reconstructs the key and uses it to establish a first connection with the quantum entropy source. This key is only used once, and afterwards it is immediately discarded.

Quantinuum’s quantum origin platform) provides certified quantum generated entropy for cryptographic use, delivering stronger classical cryptography and the highest strength post-quantum cryptography within customer’s cryptographic ecosystems. Quantum Origin’s patent-pending device independent certification mathematically proves every random number is the outcome of a quantum process without trusting the generation process before customer use.

**Figure 1:**
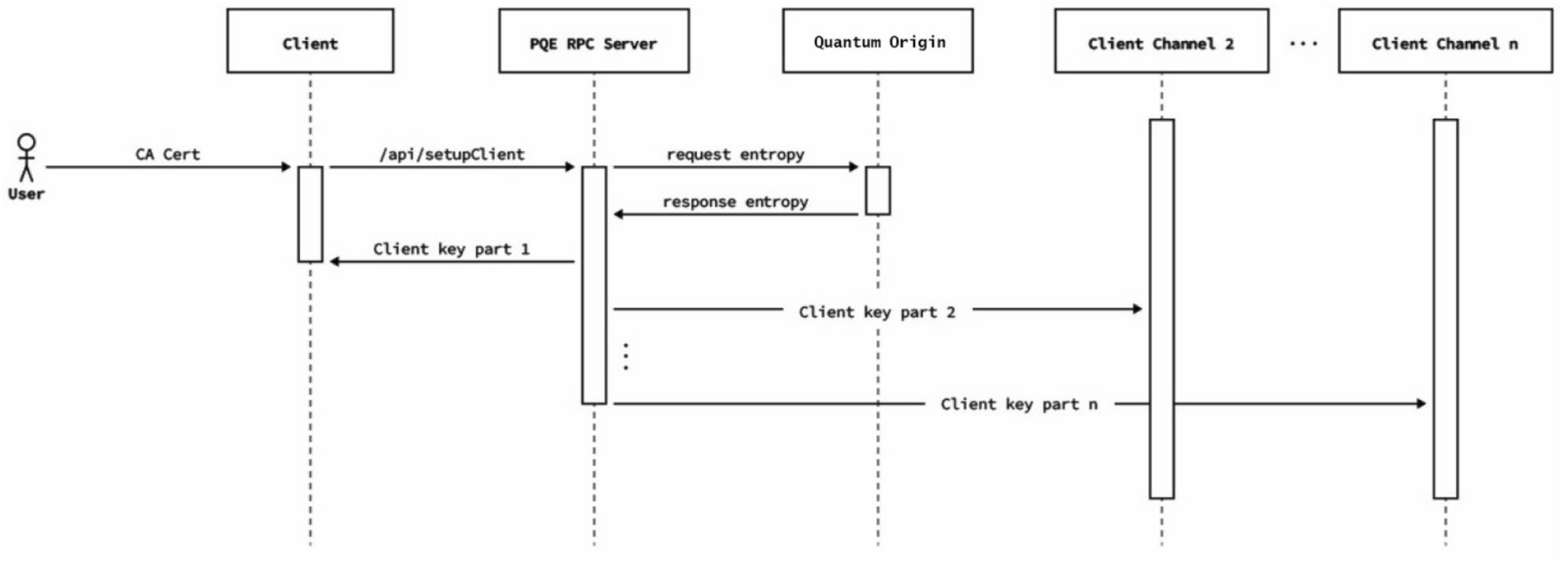
High-level schema of the first connection between the remote source of entropy and the blockchain node.

Once this first post-quantum key is used to establish the first secure connection between the LACChain node and the entropy source, they initiate a second process to renegotiate a working KEM keypair using the post-quantum algorithm, McEliece, in line with the NIST round three submissions^45^ (after NIST round four submissions, McEliece remains as a candidate for standardization^115^). This allows for the establishment of a quantum-safe connection between the entropy source and the nodes which allows the LACChain nodes to start requesting quantum entropy on demand (see Fig. 1).

### Generation of post-quantum certificates

**Figure 2:**
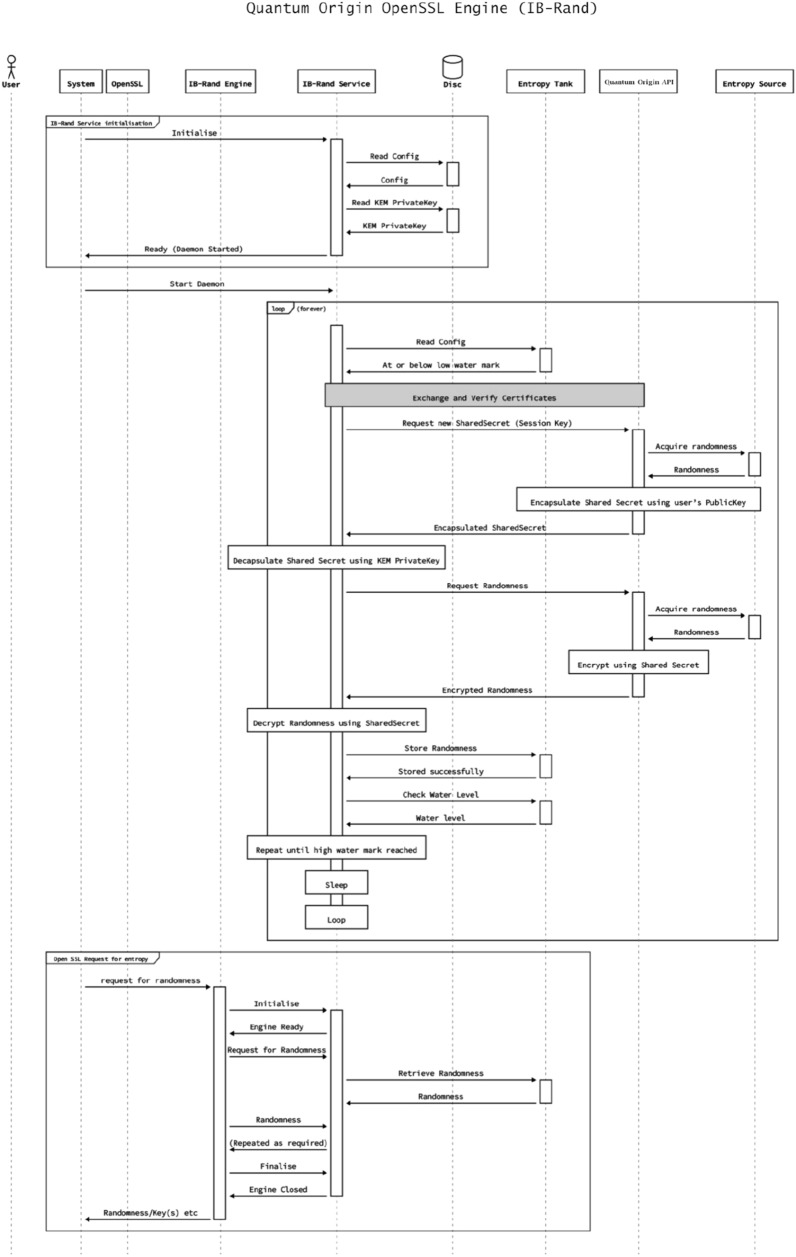
Detailed flows describing the generation and consumption of entropy on demand by the Open SSL.

Once the LACChain nodes have access to quantum entropy on demand, this entropy is consumed by OpenSSL as illustrated in Fig. 2. Permanent quantum-safe cryptographic solutions such as QKD (see “Quantum key distribution”) are not scalable today and require substantial investments in infrastructure. Feasible and practical solutions that provide quantum-resistance today involve PQC (see “Post-’quantum cryptography‘’). Instead of replacing current Internet and blockchain protocols with new ones that incorporate PQC, we tried to introduce PQC in existing frameworks.

Based on the analysis presented above, we decided to use the traditional X.509 standard, which defines an internationally accepted format for digital documents that securely associates cryptographic key pairs with identities such as websites, individuals, and organizations^116^.

By using a modified version of libSSL, the X.509 specification was extended to incorporate post-quantum and Ethereum (ECDSA) public keys, allowing blockchain nodes to use the modified libSSL to establish peer-to-peer quantum-safe channels that leverage those keys. Libssl is the portion of OpenSSL that supports TLS (SSL and TLS Protocols) and depends on libcrypto.

As discussed in “Results I—our proposal for post-quantum blockchain networks”, the nodes use the post-quantum keys to encapsulate communication with other nodes and sign transactions broadcasted to the blockchain. We decided to use the same algorithm for the generation of both types of keys (i.e., encryption keys and signing keys). Given the versatility of OpenSSL to incorporate any post-quantum algorithm, the election of the post-quantum algorithm was based on the restrictions inherent in executing blockchain transactions—essentially execution time and payload size—as different algorithms present substantial differences that condition the feasibility of on-chain verifications and storage.

**Figure 3:**
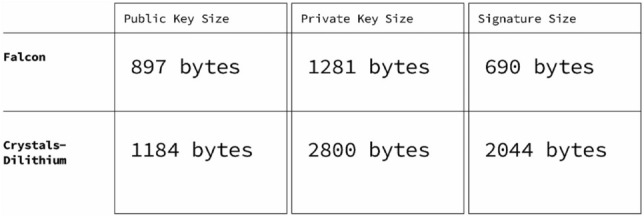
Comparison between Falcon and crystals-dilithium algorithms.

We evaluated the two finalists of the NIST competition in the signature category in round 3 submissions^45^, Crystals-Dilithium^48^ and Falcon^49^ (after round 4 submissions, NIST selected these two algorithms as recommendations in the digital signature category.). Figure 3 presents some of the differences between these two algorithms in terms of public key size, private key size, and signature size.

Both algorithms are very demanding regarding processing, memory, and amount of random material required to compute keys and signatures. However, Falcon has been acknowledged as the most compact and contains a built-in SHA3 compliant Extendable Output Function (XOF Shake256). The Ethereum VM natively supports the Keccak hashing algorithm upon which SHA 3 NIST FIPS202 is based, but it does not provide the extendable output functions (XOF) required. Further, implementing the shake XOF functionality is not straightforward.

We evaluated the other signing algorithms but speed, complexity, and the fact that we would have to implement a SHA3 compliant ecosystem for the qRNG source to feed those schemes proved Falcon to be the best option. Our solution allows for the incorporation of new post-quantum algorithms, such as those that can be standardized by organizations such as NIST in the upcoming months and years.

To use Falcon, we needed to add a new object identifier (OID), the 1.3.9999.3.1, to libSSL in order to recognize the post quantum Falcon-512 algorithm^117^.

**Figure 4:**
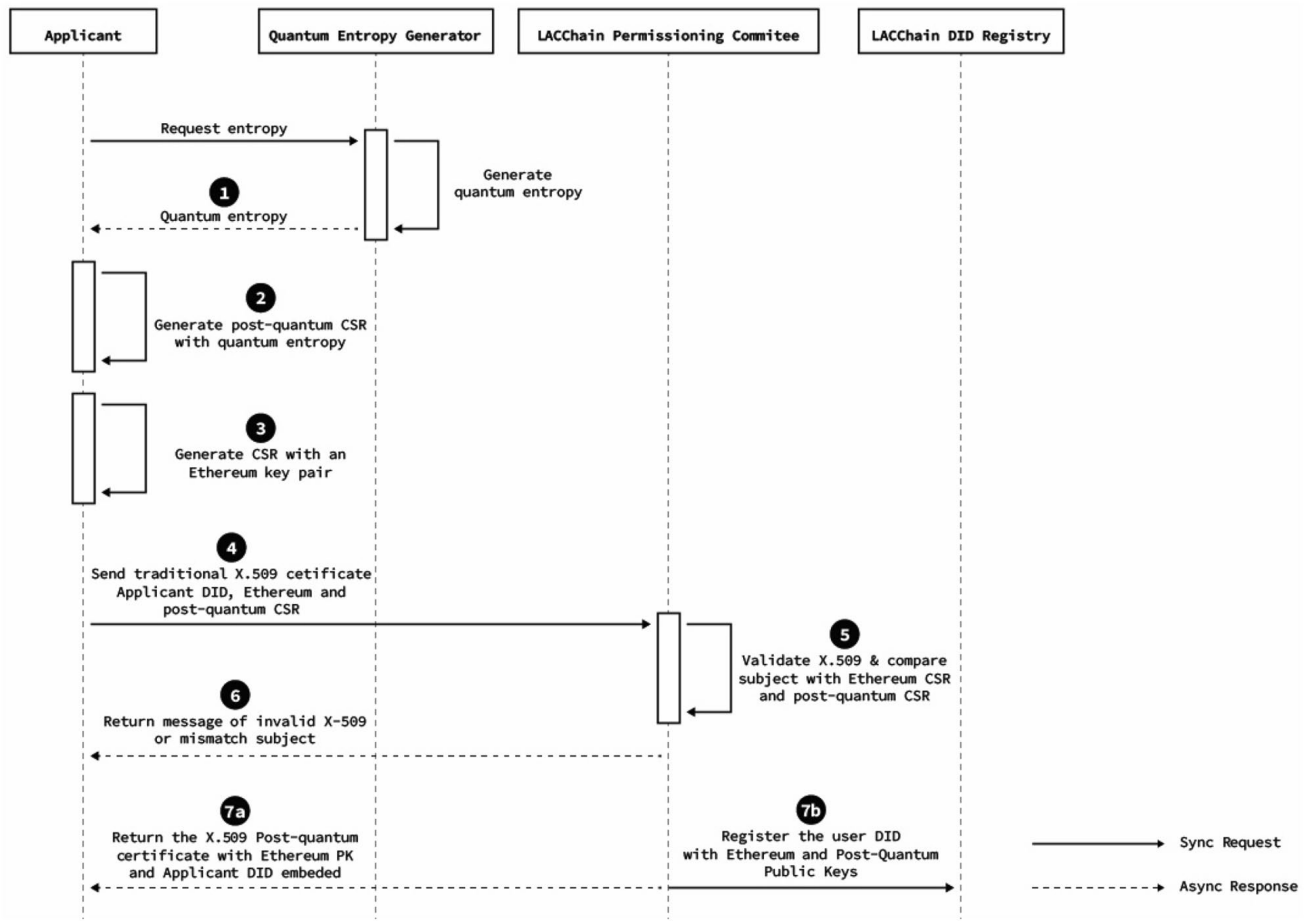
High level diagram of the post-quantum certification and on-chain registration of an entity.

**Figure 5:**
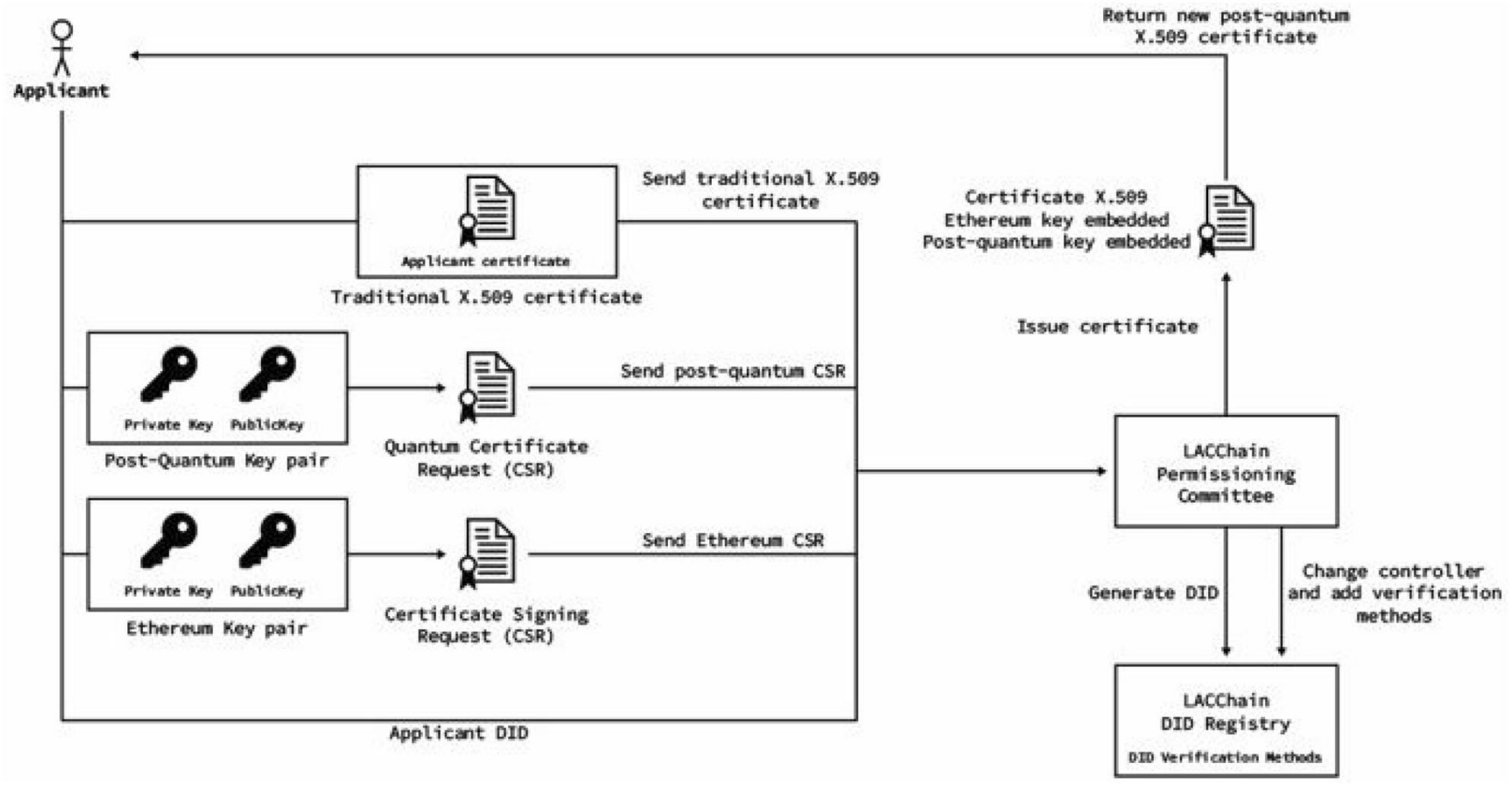
High level diagram of the post-quantum certification and on-chain registration of an entity.

The process for the generation of post-quantum certificates is summarized in Figs. 4 and 5 and broken down into the following seven steps:

- The applicant requests and receives the entropy form the qRNG as explained in section
- The applicant generates a post-quantum Falcon-512 key pair using the quantum entropy through a modified version of the OpenSLL CLI (this modification has been made by the Open Quantum Safe Initiative and we have contributed with a Debian package to simplify its installation) and builds a certificate signing request (CSR).
- The applicant generates a second CSR with an Ethereum key pair that will be used to sign transactions using the default method set by Ethereum (currently ECDSA).
- The applicant sends to a certificate authority (CA)—a role played by the LACChain Technical Team in our pilot—(i) a traditional X.509 issued by a trusted CA, (ii) a certificate signing request (CSR) for the Ethereum key, and (iii) a CSR associated for the Falcon post-quantum key.
- The CA verifies that (i) the traditional X.509 is valid, (ii) the subject in the traditional X.509 matches the subject in the CSRs, and (iii) the signature of the CSRs matches the public keys that are requested to be certified (i.e., the CSRs are valid).
- If the verification fails, the certification process is rejected, and an error message is returned to the applicant.
- If the validation process is passed, the CA proceeds to register three items into the smart contract within the blockchain called “the Decentralized Identifier (DID) Registry.” DIDs are URIs that follow a W3C standard^118^, which are suitable for the identification of individuals, entities, or other components within decentralized environments such as blockchain networks. The three items registered in the smart contract are (i) the DID, (ii) the Ethereum and Falcon post-quantum public keys, and (iii) the subject data or alternatively a proof of the subject’s identity that does not reveal subject data. Simultaneously, the CA also returns several items to the applicant, including the Falcon post-quantum X.509 certificate that contains the Ethereum public key, the Falcon post-quantum public key, and a new DID controlled by another DID derived from the ETH key.

Each of these steps is essential and additional useful clarifications are listed below:

- CSR are files of encoded text that contain information to be included in the requested certificate such as the organization name, common name (domain name), address, and country. It also contains the public key that will be included in the certificate, but the private key is not disclosed. Instead, the private key is used to sign the request so the CA can verify that the requester is indeed in control of that particular private key.
- The applicant is required to present a traditional X.509 so the blockchain CA does not have to accomplish the verification of the applicant’s identity from scratch. Both the applicant and the CA take advantage of a previous X.509 and the CA only verifies that the certified subject data in the X.509 matches the subject data in the CSRs.
- The DID Registry follows the DID standard from the W3C^118^ which presents a data model for identifiers particularly designed to be resolved and verified in decentralized registries. Every time the CA certifies a new entity, it registers the DID in the blockchain with the information about the certified Ethereum and Falcon public keys, so that anyone with access to the public blockchain ledger can resolve the entity’s DID and verify the keys associated with them. For example, this would occur when the entity is using the Ethereum key, the Falcon key, or both to sign a transaction, which will be addressed in “On-chain verification of post-quantum signatures”.

### Encapsulation of the communication between nodes using quantum-safe cryptography

Communication between nodes is made through the protocol established by the blockchain technology and varies depending on the network used. In the case of the LACChain Besu Network used for this pilot, nodes communicate via TCP and use the RLPx for data encryption (this is the same for the Ethereum mainnet, as Hyperledger Besu is an Ethereum client). This protocol seals messages with a ECDSA signature on curve SECP251k1 to link the network message to a peer address. We decided not to modify this protocol because that would require maintenance of a new blockchain technology. Instead, our goal was to keep using the Hyperledger Besu technology and develop a layer on top to make it quantum-resistant.

With the aim of developing a solution that could be used by any blockchain with any communication protocol and that would not be invasive to the protocol (i.e., does not require layer-1 modifications), our solution consist of adding a point-to-point TLS tunnel modified to support post-quantum keys where the post-quantum X.509 certificates described in “Generation of post-quantum certificates” are used for identification and authorization.

**Figure 6:**
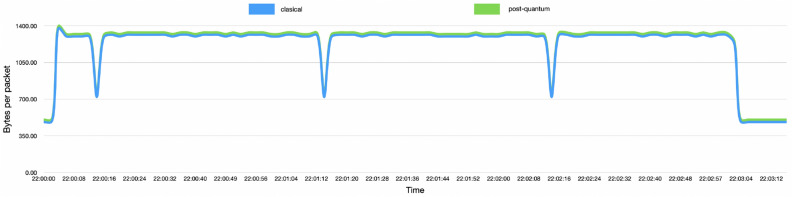
Bytes per package.

In order to evaluate the overhead of the communication between nodes using this post-quantum channel, we measured the bytes per packet that travel between nodes with and without the post-quantum channel. As presented in Fig. 6, there is a constant overhead of 22 bytes introduced by the post-quantum signature. This is almost negligible and does not represent a challenge for the adoption of this solution. It could even be possible to use other post-quantum algorithms with larger key lengths. In “Performance results” we also present an analysis of the overhead in CPU and memory consumption of the overall implementation with the post-quantum channel and the verification of post-quantum signatures described in “EVM pre-compiled-based signature validation support”.

Once this tunnel is established, each node must route the traffic aimed at its counterpart through the TLS tunnel, making it unfeasible for a quantum computer to intercept the traffic and impersonate a node. This protects the blockchain network from different types of attacks. For example, because we are not modifying the blockchain protocol in our permissionless network, the node producers that vote for the generation of new blocks are still materializing this vote in an ECDSA signature (the consensus protocol requires 2/3+1 of node producer’s signatures for a block to be considered valid) that is neither replaced not complemented with a post-quantum signature. However, if a hacker was to discover all the private ECDSA keys of the validator nodes and tried to tamper with the block production by changing the valid transactions and use the validator nodes’ signatures to sign them, it could not achieve it because it cannot intercept the communication between nodes where they could provoke this type of man-in-the-middle attack. The hacker would need to hack and access each of the validator node servers, for which quantum computers present no advantage.

In any case, despite the fact that we believe this threat is removed with our solution, it would be easier and more convenient to modify the Ethereum protocol so cryptographic algorithms different from ECDSA, such as Falcon-512, are recognized and can be used by validator nodes to sign blocks.

### Signature of transactions using post-quantum keys

Unlike the first three phases, the implementation of the fourth phase requires us to be particular about each specific blockchain network. There are blockchain protocols that recognize different encryption algorithms and/or are already flexible in incorporating new ones. At the present moment, this is not the case of Ethereum and the Ethereum-client, Hyperledger Besu, on top of which the LACChain Network used in the pilot is built^59^. In this context, our way for introducing a mechanism to add a quantum signature to the transactions broadcasted to the network without modifying the blockchain protocol was the development of a relay signer and a meta-transaction signing schema.

A meta-transaction is a mechanism through which to wrap a regular transaction into another transaction addressed to a method of a smart contract (a.k.a. relay Hub) which unwraps and executes the original transaction. Because the meta-transaction is a regular call to a smart contract, we can add new parameters along with the original transaction. In this case, our design allows us to add the writer node’s URI (a DID^118^) and a post-quantum signature to the original transaction.

**Figure 7:**
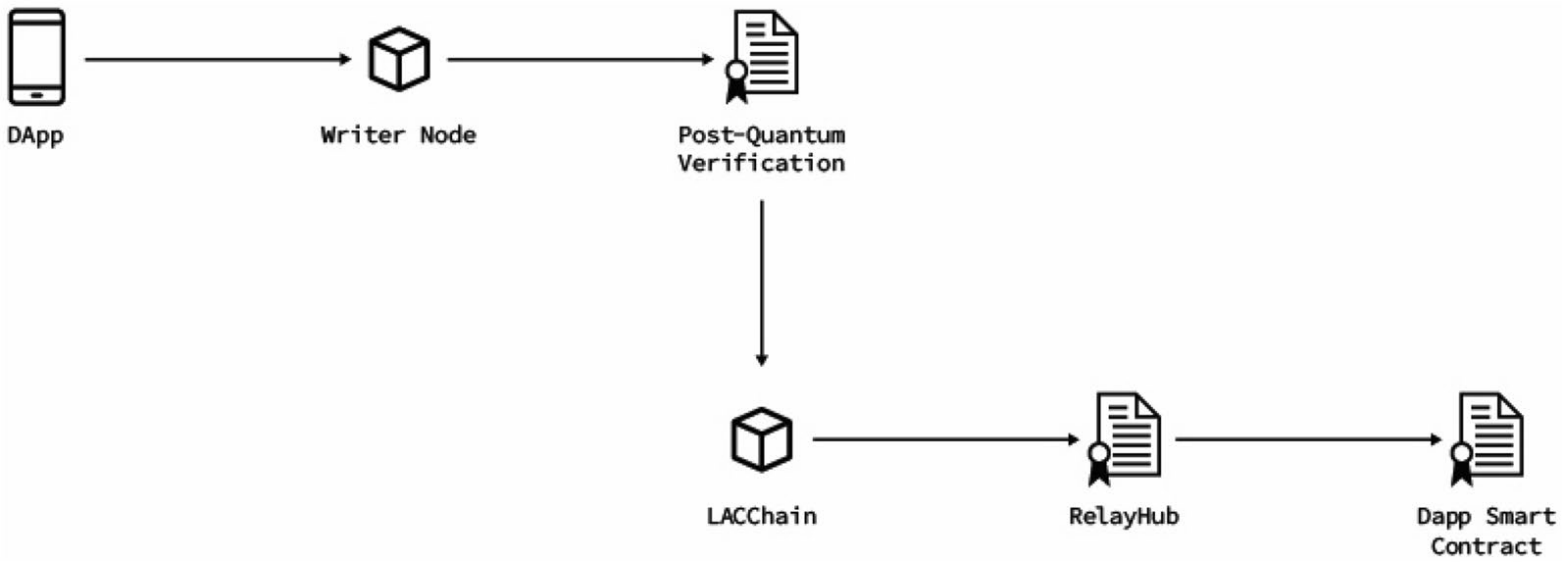
High level diagram presenting the different components from the DApp (it can also be an app or any application connected to the writer node and generating transactions) and the smart contract that it is calling.

**Figure 8:**
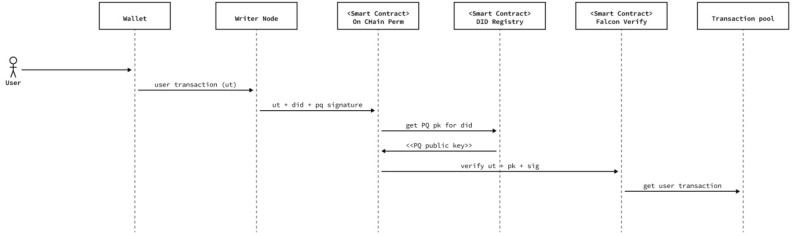
High level diagram illustrating the flows from the generation of a transaction to the incorporation of that transaction to the transaction pool of a node, after validating the post-quantum signature.

We have developed a relay signer that is provided to the writer nodes -the only nodes allowed to broadcast transactions according to the LACChain topology^119^- that can manage post-quantum keys. This component exposes a JSON-RPC standard interface, instrumenting methods to make the whole operation transparent to the user. Each writer node is responsible for keeping its Falcon-512 private key safe, and the signer to generate the meta-transaction. Figure 7 summarizes these concepts. Furthermore, full interaction among components is presented in Fig. 8.

Following the EIP-155^120^, signatures in Ethereum take nine RLP encoded elements: nonce, gasprice, startgas, to, value, data, chainid, 0, 0. For consistency, we took the same stream of data to generate the Falcon-512 signatures. This guarantees the integrity of the original transaction -the writer node cannot modify it- and its quantum resistance by adding the post-quantum signature in the meta transaction. Writer nodes leverage the post-quantum public keys certified by a CA in the post-quantum X.509.

It is worth mentioning that we are only adding a post-quantum signature in the meta transaction that is created by the writer node, but original senders (i.e., blockchain addresses) are still using only the ECDSA signatures to sign their transactions. Ethereum addresses are the 20 bytes of the SHA3 hashed ECDSA public key, so the public key is not directly exposed. However, when an address sends a transaction, the private key is used to sign it and therefore it is necessary to reveal the public key so the transaction can be verified.

Thus, if a blockchain address is in possession of certain tokens or has a particularly relevant role in the network (e.g., being permissioned in a smart contact that can issue digital bonds), a quantum computer could be used to hack the private key associated to that address and send transactions to the blockchain that impersonate the true owner. This would allow the hacker to steal the victim’s funds or to assume their particularly relevant role in the network, respectively.

Our solution allows to remove this threat by enabling each smart contract to require post-quantum authentication and leveraging for it one of our on-chain verification mechanisms presented in “On-chain verification of post-quantum signatures”. Only the transference of Ether would not be protected, but LACChain does not have Ether enabled.

As in the case of the signatures by validator nodes described in “Encapsulation of the communication between nodes using quantum-safe cryptography”, it would be much easier, ideal, and convenient to have the Ethereum technology enabling the use of quantum-safe cryptographic algorithms that can be used at the protocol level to sign and verify transactions. We believe that Ethereum Improvement Proposals (EIPs) such as the EIP-2938^121^ are moving in the right direction and are very aligned with the work described in this paper.

### On-chain verification of post-quantum signatures

When a writer node adds a post quantum signature to the meta-transaction and broadcasts it to the network, there must be a mechanism for the signature to be verified. In the regular Ethereum protocol, there is not explicit verification for any signature. In the Ethereum protocol, for a given ECDSA signature, an address is derived and used as the identity of the person willing to execute and pay for a blockchain operation. For the LACChain Besu Network, we have decided to implement a verification protocol based on the Onchain Permissioning feature, which is based on smart contracts. This feature enables each node to intercept every transaction and run different validations before incorporating them into their transaction pool and replicate them to their peers.

Particularly, according to our protocol, nodes use the post quantum signature to verify the authenticity and integrity of the transaction. As the name of the feature implies, this is resolved by making a local call to a smart contract existing in the network, which receives several parameters (sender address, target address, transaction value, gas price, gas limit, payload). To our purpose, nodes check the “target address” and dissect the “payload”, as described below.

As previously discussed (see “Signature of transactions using post-quantum keys”), we use a meta-transaction model for executing user requests. This means that there is a single-entry point for our network, which is the address of the Relay Hub contract where the meta-transaction is directed. Therefore, the first Permissioning check consists of verifying that the target address is the Relay Hub contract. Otherwise, nodes will reject the transaction.

Once the Relay Hub smart contract has been verified as the target of the transaction, each node extracts the original payload transaction, the writer node’s DID, and the Falcon-512 signature from the original transaction in order to verify the signature. Additionally, a call to the DID Registry allows for retrieval of the public keys associated with it, including the post-quantum public key that should match the post-quantum signature. With this information, each node receiving a transaction from a peer takes the original transaction, the public key, and the signature, and verifies their consistency. If it is not consistent, they reject the transaction (i.e., they do not add it to their transaction pool, nor propagate it to other peers).

To summarize, the protocol we have designed consists of three steps:

1. Every node that receives a meta-transaction -from the node that created it or from another node that replicated it- checks the sender. This involves obtaining the DID from the meta-transaction and locally querying the DID Registry in order to resolve (i.e., obtain) its Ethereum keys (ECDSA). They then verify that the public key derived from the ECDSA signature of the meta-transaction has control over the node’s DID that generated it.
2. If Step 1 is successful, the node calls the DID Registry again and now resolves the post-quantum public key associated with the DID as well as the Ethereum public key verified in Step 1.
3. With the post-quantum public key resolved from the DID Registry in Step 2, the post-quantum signature, and the original transaction, each node then verifies the post-quantum algorithm.

If the three previous steps are successfully completed, nodes add the meta-transaction to their transaction pool and replicate them onto other nodes so that the validators will receive them and add them into the next block.

As previously stated, we have chosen Falcon-512 as our post-quantum algorithm. There is not yet an ideal way of implementing the Falcon-512 verification required to accomplish the Step 3 of this verification process nor any other post-quantum algorithm, in Ethereum-based networks. We have developed three alternative mechanisms and analyzed their pros and cons, which are presented in detail in “Comparison between different solutions for verification of post-quantum signatures”.

These three mechanisms are:

- Implementing the verification code in Solidity (see “Verification code in solidity”).
- Implementing solidity instruction in the Solc compiler and corresponding EVM opcode, written in Java (Besu is written in Java), that performs a call through JNI to a NIST-compliant and high performance native Liboqs library outside of the EVM virtualized environment (see “EVM virtual machine-based signature validation support”).
- Refactoring the EVM opcode Java from the EVM virtual machine into a pre-compiled contract (a EVM Java-code native smart contract) that performs the call through JNI to the NIST compliant, high performance native Liboqs library outside of the EVM virtualized environment (see “EVM pre-compiled-based signature validation support”).

We hope that in the not-so-distant future, we can use this effort in alignment with the upcoming protocol changes in the form of the Accounts Abstractions, which will allow us to replace ECC cryptography with new algorithms, including post-quantum.

#### Verification code in solidity

The natural execution environment for the blockchain is the Ethereum Virtual Machine; thus, in our first attempt, we implemented the verification code entirely in the Solidity language. We dissect the reference implementation in the following modules and discuss the implementation of the highlighted functions one by one.

**Figure 9:**
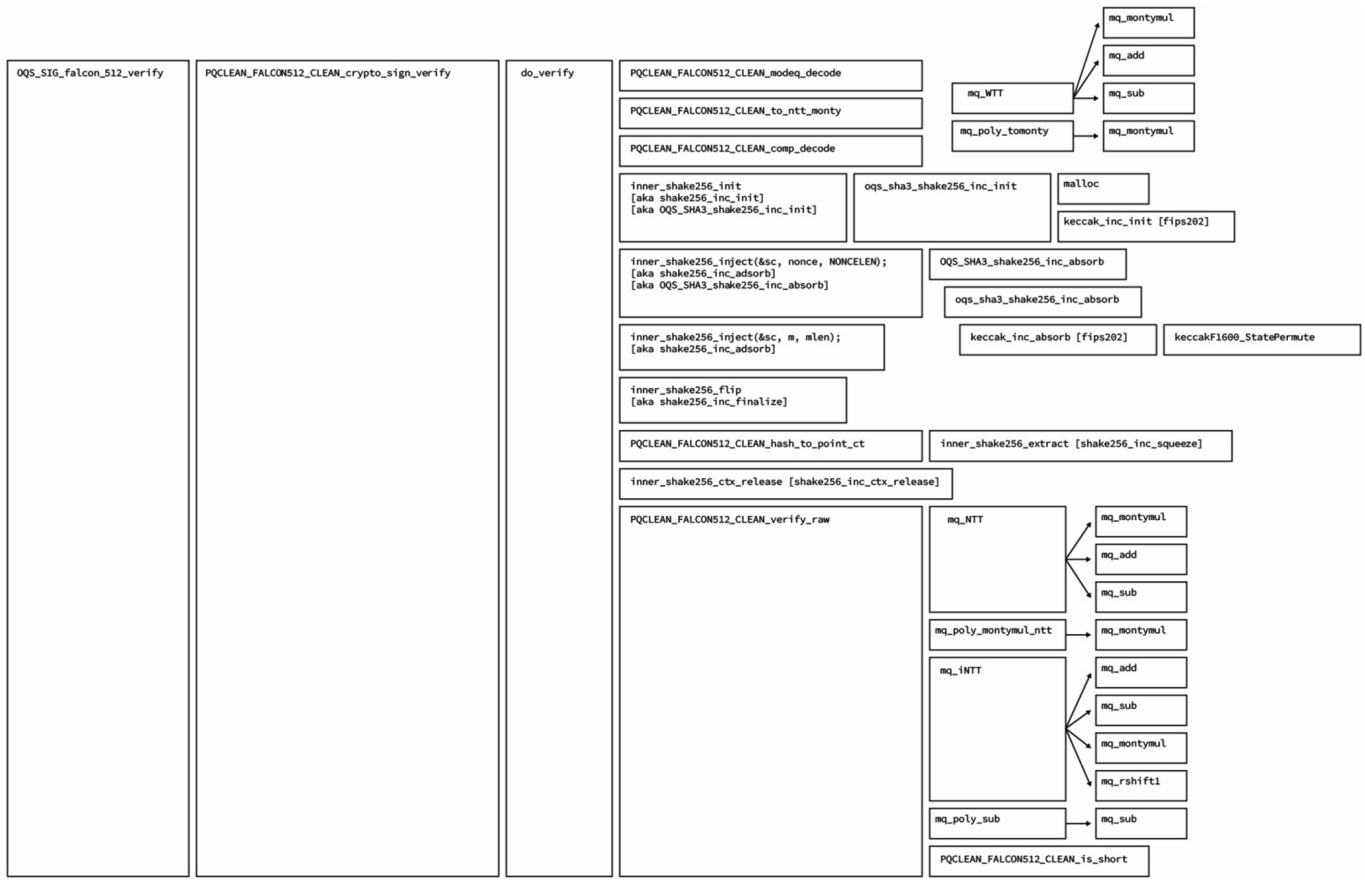
High level function hierarchy of Falcon highlighting the necessary calls for verification.

**Figure 10:**
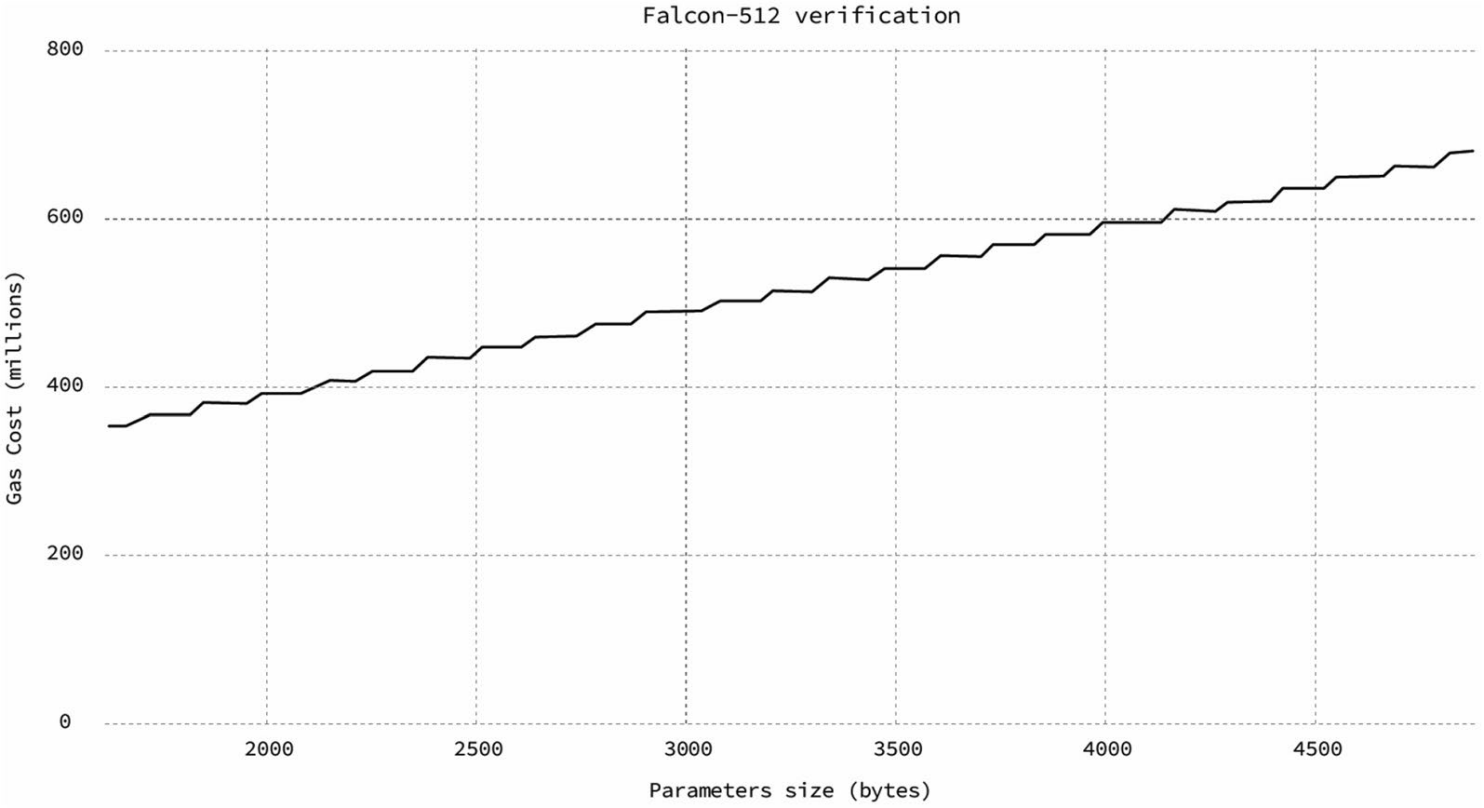
Gas consumption by the on-chain verification of Falcon-512 using the Solidity smart contract.

Implementing the highlighted portions of Fig. 9 in Solidity allowed for on-chain signature verification. Upon the completion of the development process, we faced two major problems. The first problem was the code size. It exceeded the 24kb limit that Ethereum mainnet imposes. This limit could have been exceeded in LACChain because LACChain has different boundaries, but such large code sizes are not ideal. The second and more major problem was the execution cost. In Fig. 10, we present a chart with the execution cost of the verification of the known answer tests provided by the Falcon implementation. If we compare the average 500 million gas units for a single Falcon signature verification, with the current block limit of 12 million gas units in the Ethereum mainnet, we can conclude that this approach is completely impractical at this point.

Our implementation of post-quantum signatures using Solidity code is the first one that has been developed to our knowledge. The open-source code can be found at^122^.

#### EVM virtual machine-based signature validation support

An EVM based approach requires modification of both the Solidity compiler (solc) and the Ethereum Virtual Machine (EVM) that underpins the Besu Hyperledger technology used by LACChain.

These changes are applicable across all Ethereum-based networks but require all participating nodes within the blockchain to utilize the updated solidity compiler and EVM. The Java Native Interface (JNI) is also required in addition to ensuring that compatible OpenQuantum Safe (an open-source venture) Liboqs libraries are installed. Performance is therefore limited only by the native liboqs library and the native node processing power.

The solidity modification is minor, and only requires adding an instruction token to the existing instruction list. The modification to the EVM is similarly minor and only requires adding a Java class to a Falcon Verify operation and registering the class with the operations available for that version of the EVM virtual machine. This implementation provides a simple Gas cost of 1. However, an extended example could be made to utilize the memory-block size cost calculation performed by SHA3.

**Figure 11:**
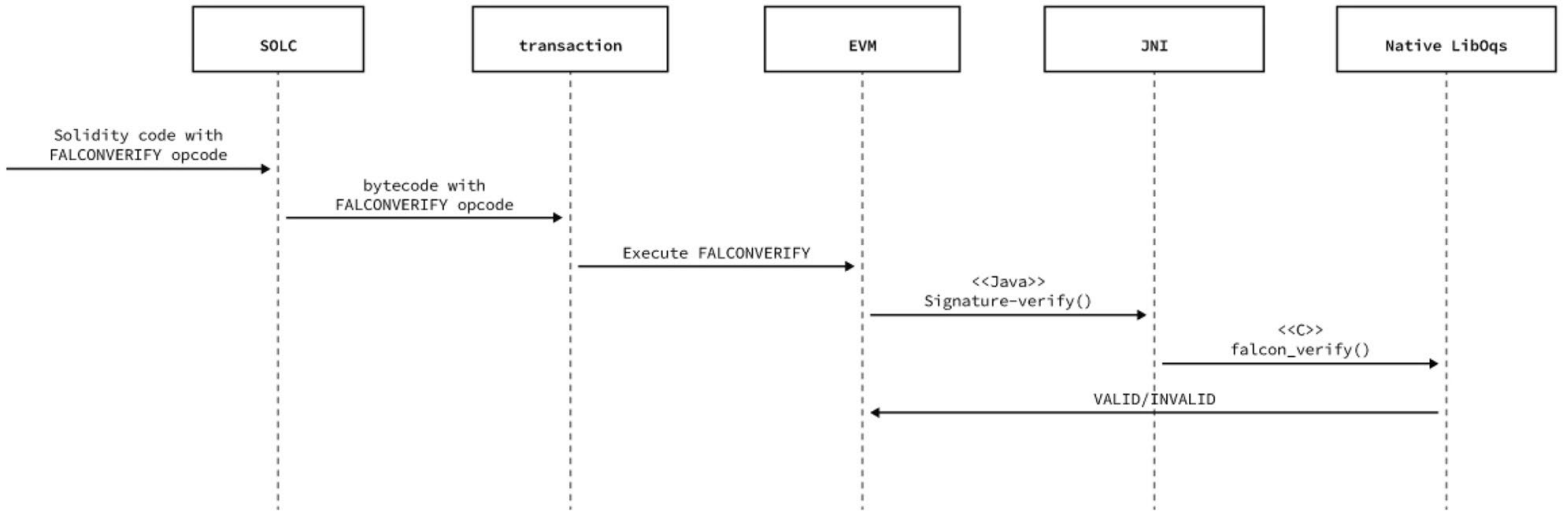
EVM virtual machine-based signature validation support.

**Figure 12:**
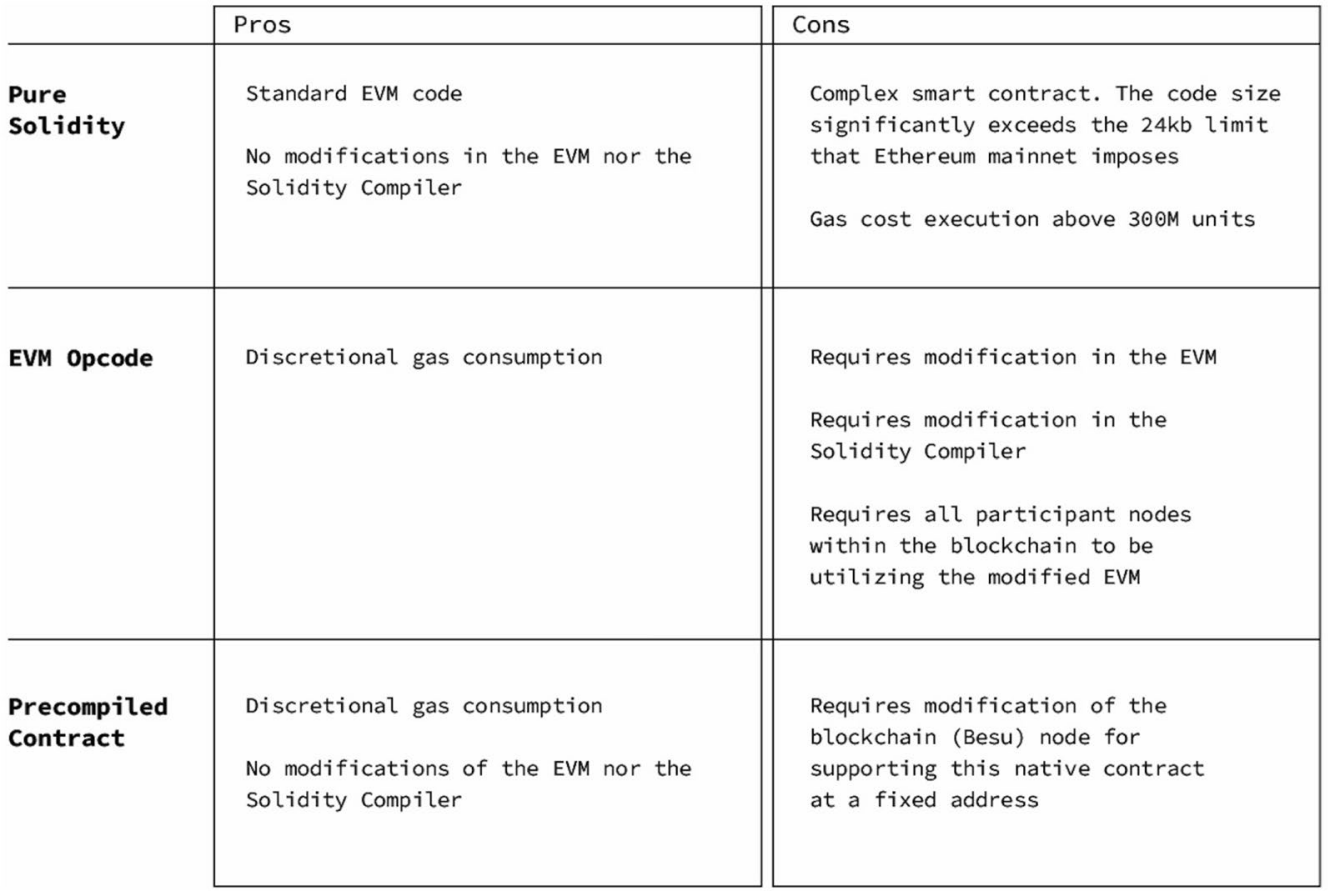
Pros and Cons of Pure Solidity, EVM Opcode, and Precompiled contract.

The approach only uses one opcode from the 6000 opcodes limit call within the standard configuration of Ethereum. The real-world performance of the signature verification is as fast as the hardware can perform—aligning with the performance observed by the OpenQuantum Safe teams.

The utilization of the OpenQuantum Safe liboqs library ensures minimal operational delay or risk in maintaining updated quantum algorithms in line with NIST and the OpenSource Safe current standards. The Java class implemented for the EVM can also be extended beyond Falcon-512 and to allow Falcon-1024 or other signatures.

The EVM stack word width is 256 bits, which naturally fits with the existing 256-bit hashes used in the classical encryption. However, post-quantum signatures with larger memory requirements will become less optimal unless the stack word width is increased at the cost of compatibility with previously operational blockchains. Finally, the POC EVM implementation utilizes Falcon-512, which minimizes this impact while also providing a security level that is in alignment with classical AES-256. Figure 11 summarizes the interactions described in this subsection.

#### EVM pre-compiled-based signature validation support

The pre-compiled approach transplants the EVM falcon verify operation Java class into a EVM precompiled smart contract (a native Java compiled smart contract). This approach has two benefits that reduce operational impact:

- No change to the Solidity compiler.
- No change to the underlying EVM virtual machine.

This facilitates the distribution of the quantum signature verification separate from the compiler and EVM releases. The approach therefore brings all the benefits of the EVM opcode implementation but with less operational work. The JNI and Liboqs libraries are used identically, offering speed and ease of maintenance. It is also worth mentioning that given this verification is meant to be executed before a node joins the blockchain, it could easily be replaced in the future without affecting the consensus. It will only be necessary to modify the deployment scripts.

Our implementation in the LACChain Besu Network proved the feasibility of this approach. Using the post-quantum channel described in “Encapsulation of the communication between nodes using quantum-safe cryptography” and the EVM pre-compiled-based signature validation, the use of memory in the node presented an increase from around 150 megabytes to around 200 megabytes, with minor variations based on the number of transactions executed. In terms of CPU consumption, the post-quantum scenario presents an overhead of 10% to 30%. The results are presented in “Performance results”. Unlike the on-chain verification using a Solidity smart contract described in “Verification code in solidity”, these performance metrics show that the pre-compiled smart contract provides a scalable path to secure transactions and protect blockchain assets from attacks by quantum computers.

Implementing this solution in the LACChain Hyperledger Besu Network required changes in the protocol with respect to other Ethereum networks, including the Mainnet. This is against our goal to preserve compatibility with the Ethereum community. Therefore, the ideal way to proceed with this third approach for the verification of Falcon signatures is submitting an EIP for the community to evaluate the incorporation of a pre-compiled smart contract into the Ethereum protocol, for the community to evaluate and decide to move together in this direction.

#### Comparison between different solutions for verification of post-quantum signatures

The three alternatives that were designed and tested for the verification of post-quantum signatures are successful for verification but either are not scalable or require substancial modifications in the blockchain network. The Solidity native implementation presented in “Verification code in solidity” is not scalable due to the amount of gas required for the execution of the code, although it does not require a modification of Besu or Ethereum. The modification of the Solidity compiler and the EVM, as well as the pre-compiled smart contract (presented in “EVM virtual machine-based signature validation support” and ’‘EVM pre-compiled-based signature validation support‘’ respectively) are computationally scalable. However, they require undesired modifications unless otherwise agreed upon by the entire Ethereum community, which is the goal we aim at to pursue in the next step of this implementation.

Additionally, the solutions described in “EVM virtual machine-based signature validation support” and ’‘EVM pre-compiled-based signature validation support‘’ use the Java Virtual machine. However, unlike the Solidity native implementation, these two techniques are not impacted by EVM or JavaVM mathematical computational problems maintaining validity and security between releases. Instead, the pure C native method of Liboqs implements its own mathematical validity tests as part of the C build system. The result is that regardless of Java or EVM release, the verifying Liboqs library remains mathematically valid (assuming no optimizations or changes that invalidate tests). This approach allows organizations to separate security requirements, offering more precise maintenance and governance. However, this approach would require extra security protocols with the additional overhead. Figure 12 shows some advantages and disadvantages of Pure Solidity, EVM Opcode and precompiled contract.

### Performance results

In this subsection we present the results of the verification of post-quantum signatures following the preferred approach, which was described in ’‘EVM pre-compiled-based signature validation support‘’. The environment where the tests were performed is the following:

- Server type: virtual
- Environment: Google Cloud
- Locations: us-west2-b and us-east1-b
- CPU: 2vCPUs
- Memory: 7.5GB
- Besu version: 23.1.2
- Java version: openjdk 17.0.6

We prepared classical and post-quantum configurations of the LACChain Besu Network and performed 3-min interval tests to evaluate the overhead in the use of memory and CPU expected in the post-quantum configuration.

**Figure 13:**
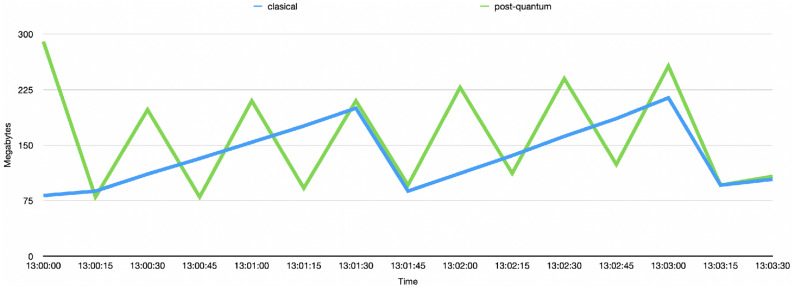
Comparison between the use of memory in the classical and post-quantum scenarios when sending 5 tx/s.

Figure 13 depicts the behavior of the Java memory when sending 5 tx/s in both the classical and post-quantum scenarios. In the classical scenario, the memory reaches peaks up to 206 Mb with 2 memory releases. In the post-quantum scenario, the peaks reach up to 256 Mb and the releases are more frequent happening 6 times in the 3-min interval. Because the Besu nodes use Java as the programming language, the analysis of the Java memory consumption are extensible to the node memory consumption.

**Figure 14:**
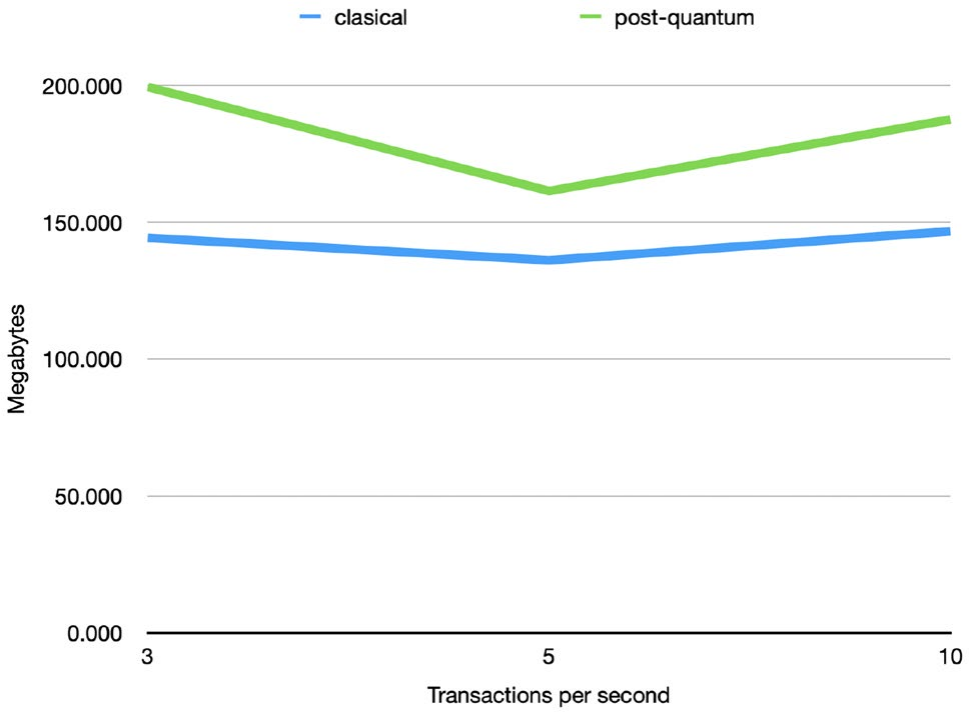
Comparison between the average use of memory in the classical and post-quantum scenarios when sending 3, 5, and 10 tx/s.

Figure 14 depicts the average use of Java memory when sending 3, 5, and 10 tx/s. The difference does not depend on the number of tx/s and remains relatively stable. In the classical scenario, the memory consumption oscillates between 136 Mb and 147 Mb while the post-quantum scenario presents a memory consumption between 162 Mb and 199 Mb.

**Figure 15:**
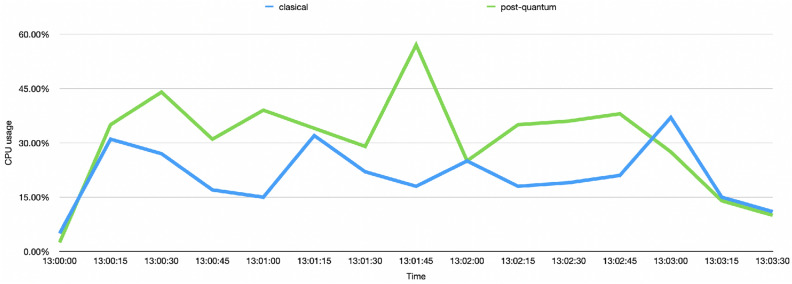
Comparison between the use of CPU in the classical and post-quantum scenarios when sending 5 tx/s.

Figure 15 depicts the behavior of the CPU consumption when sending 5 tx/s in both the classical and post-quantum scenarios. In the classical scenario, the memory reaches maximum CPU consumption peaks between 30% and 35% and averages 21%, while the classical scenario reaches maximum CPU consumption peaks between 40% and 60% and averages 30%.

**Figure 16:**
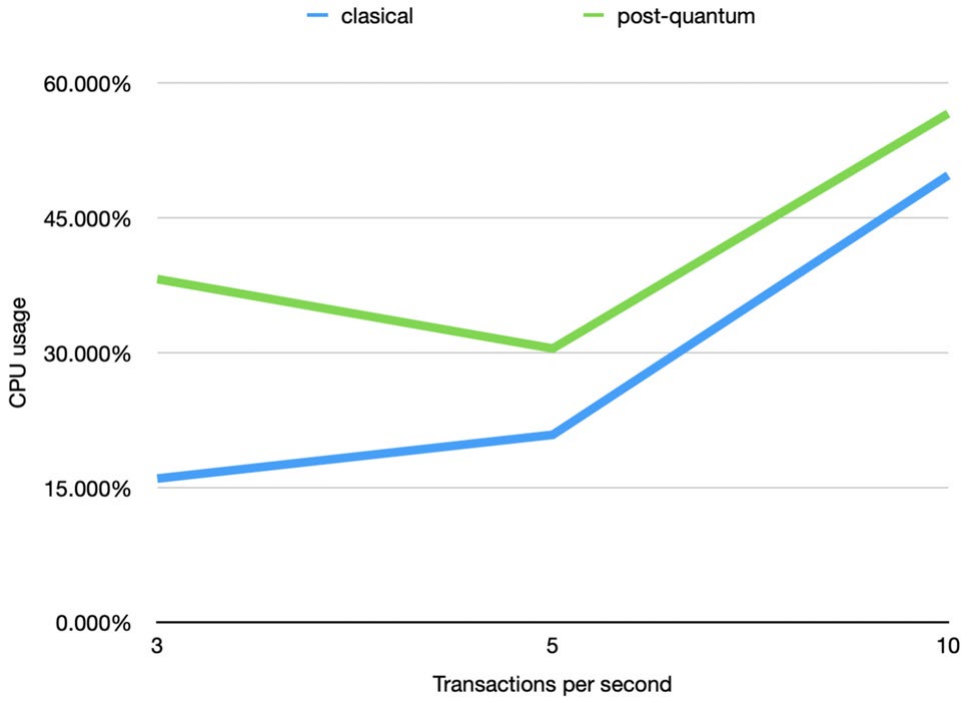
Comparison between the average use of CPU in the classical and post-quantum scenarios when sending 3, 5, and 10 tx/s.

Figure 16 depicts the average CPU consumption when sending 3, 5, and 10 tx/s. The difference does not depend on the number of tx/s but grows with the number of tx sent. In the classical scenario, the CPU consumption oscillates between 16 and 50% while the post-quantum scenario presents a CPU consumption between 38 and 57%.

## Discussion

We have analyzed the various areas of blockchain technology threatened by the advent of quantum computers and identified two areas that are under particularly critical risk: internet communication between blockchain nodes and the blockchain transaction signatures that allow to protect assets and value stored in the ledgers. Today, the most popular blockchain protocols rely on algorithms such as ECDH and ECDSA, which are susceptible to attacks by quantum computers. Current quantum computers have already proven themselves able to break short asymmetric keys using Shor’s algorithm and it is only a matter of time before robust quantum computers currently under development will be able to break larger and larger keys. As the “hack today, crack tomorrow” motto warns, quantum computers will be able to access secrets retroactively. This is particularly critical for blockchain, where information is recorded publicly and immutably so having access to all the information any time in the future will not even require any hacking. Quantum computers can also hack assets stored in blockchain netwoks, which add up to hundreds of billions of dollars today and continue to grow. If these assets are not protected from quantum computers in time, a very critical global financial crisis could happen.

We presented a detailed analysis of related work, which is mostly theoretical. Proposals for quantum-resistant blockchain can be classified into quantum blockchain networks -which rely on QKD and entanglement—and post-quantum blockchain networks -which rely on post-quantum cryptography. In this paper, we have proposed an end-to-end framework for post-quantum blockchain networks and we have implemented it in an EMV-compatible (i.e., Ethereum-based) blockchain network. Our implementation is the first robust and scalable solution to protect communications and signatures in an EVM-compatible blockchain network from attacks by quantum computers. Our solution has responded to the critical challenge of protecting existing assets in blockchain networks.

Our solution consists of modifying libSSL to incorporate post-quantum algorithms that are quantum-resistant and adding post-quantum keys into X.509 certificates derived from traditional certificates. The nodes use these post-quantum X.509 certificates to encapsulate their communication by establishing post-quantum TLS tunnels. The nodes also use the post-quantum key associated with the certificate to sign the transactions they broadcast to the network. Additionally, in order to guarantee pure random keys, we have used Quantum Origin) as a qRNG. We have implemented this solution in the LACChain Besu Network, which is built on Ethereum technology. Our framework can be applied to most blockchain networks and our implementation could be use to bring quantum-resistance to other EVM-compatible blockchain such as Ethereum Mainnet.

There are several strengths and benefits to our implementation. Firstly, it uses a quantum source of entropy (i.e., a non-deterministic quantum random number generator) as the seed for the generation of post-quantum keys. Secondly, we have achieved quantum resistance in communications between nodes using a post-quantum scheme that does not required QKD networks still under development. Our implementation of post-quantum TLS tunnels between blockchain nodes is the first one to date. Thirdly, we have incorporated a Falcon-512 post quantum signature in every transaction that is required by the network for transactions to be valid, which allows to secure the hundreds of billions of dollars in assets and value stored in existing blockchain networks. As we do no replace the original ECDSA signature, upgrading the network to achieve quantum-resistance is much feasible. Fourthly, we have proposed three different alternatives for the post-quantum signature verification, which every node accomplishes before adding a transaction to the transaction pool and replicating it. Therefore, if a signature is not valid, the transaction is never propagated nor added into a block. Our implementation of verification of Falcon-512 signatures in Solidity smart contracts is the first one to date.

The three different solutions for the verification of the post-quantum signatures that we have proposed, developed, and tested are: an implementation of the verification code in solidity, the addition of a new operation code into the EVM assembly language (with a corresponding Solidity compiler modification to generate this _opcode_), and the introduction of a new pre-compiled (i.e., native) smart contract. These three implementations are focused on ensuring the minimization of the number of operations and amount of entropy required, in addition to being NIST compliant. The first solution, despite the fact that it is totally compatible with the current protocol, is not computationally scalable due the enormous gas cost it involves. The latter two were implemented through a native Liboqs library outside of the EVM runtime allowing us to improve the execution time and to adjust gas consumption. The experience gathered through this work will lead our team to raise the discussion through an EIP to support the use of Falcon-512 for on-chain verifications. This is the way to not diverge LACChain or any other particular blockchain network from Ethereum consensus and, at the same time, improve the security of any implementation of the protocol.

In addition to the potential modifications of the Ethereum protocol to enable our layer-two implementation, we also believe it is necessary to modify current blockchain protocols to introduce new post-quantum signature cryptographic algorithms that allow the use of post-quantum cryptography natively. We hope that our work can contribute to current efforts in this direction such as the EIP-2938.

With respect to other blockchain networks that are not EVM compatible (i.e., Ethereum-based), the framework for a post-quantum blockchain network presented in this paper is applicable too. However, the implementation will vary based on the technology used. Therefore, this solution might enable quantum-resistance in other blockchain networks in a more efficient way than in the Ethereum-based network.

As previously stated, it could be argued that by the time large quantum computers capable of breaking current cryptography are ready, blockchain protocols will have upgraded their cryptography to post-quantum safe algorithms. However, considering that blockchain networks are immutable ledgers, the rule of “hack today, crack tomorrow” urges us to protect them now, or at least to have a plan and a roadmap for it. None can predict exactly when will quantum computers be large and robust enough to hack blockchain networks but it is very likely that quantum adversaries will not publicly disclose having them. Instead, they will try to use them silently to go undetected when carrying out attacks.

## Data Availability

The datasets generated during and/or analysed during the current study are available from the corresponding author on reasonable request.
